# Projection imaging of myocardial perfusion: minimizing the subendocardial dark-rim artifact

**DOI:** 10.1186/1532-429X-14-S1-P275

**Published:** 2012-02-01

**Authors:** Behzad Sharif, Rohan Dharmakumar, Troy Labounty, Chrisandra Shufelt, Louise Thomson, Noel Bairey Merz, Daniel S Berman, Debiao Li

**Affiliations:** 1 Biomedical Sciences, Biomedical Imaging Research Institute, Cedars-Sinai Medical Center, Los Angeles, CA, USA; 2 Bioengineering, UCLA, Los Angeles, CA, USA

## Summary

We demonstrate that projection imaging of first-pass myocardial perfusion is robust to a major cause of the so-called subendocardial dark-rim artifact (DRA); thereby proposing radial k-space sampling as the preferred acquisition scheme for DRA-free perfusion imaging.

## Background

Current methods for clinical myocardial perfusion (MP) imaging suffer from image artifacts, specifically the so-called subendocardial dark-rim artifact (DRA) [1-3]. DRAs are especially limiting since they can reduce the sensitivity/specificity of detecting subendocardial MP deficits; hence, eliminating such artifacts remains an active area of research [1]. In this work, we demonstrate that projection imaging of MP is free of Gibbs ringing [2], a major cause of DRAs for conventional schemes.

## Methods

**Figure 1:**
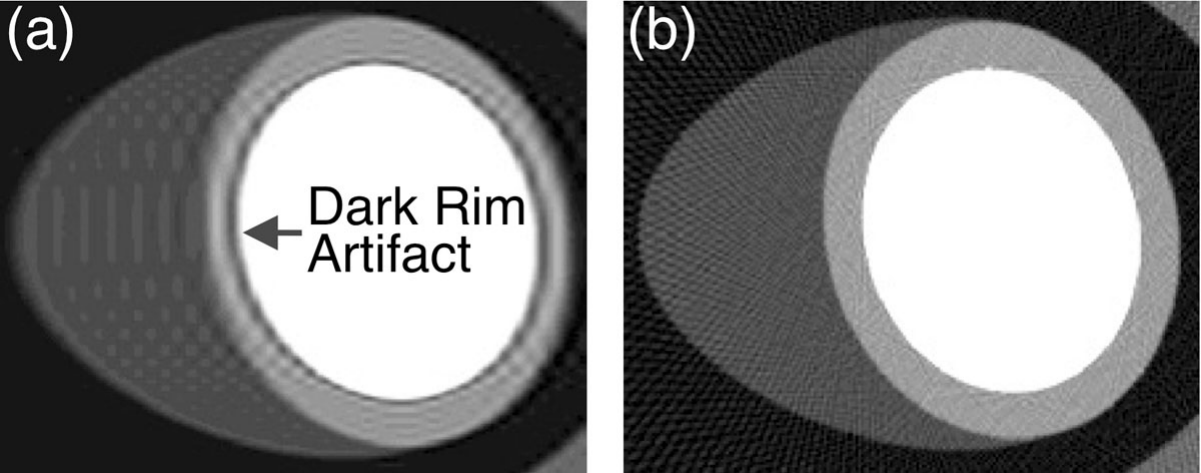
Numerical simulation (with realistic intensity values) demonstrating robustness of projection imaging to Gibbs ringing. (a) Cartesian imaging with 108 PEs (DRA is seen as highlighted); (b) projection imaging with 108 projections and filtered backprojection reconstruction (mild streaking artifacts are present).

In Cartesian MP MR, Gibbs ringing along the phase-encode (PE) direction leads to DRAs due to an inherent property of the Fourier transform (FT) at sharp signal intensity transitions [2]. However, in projection imaging, the underlying data transform is the Radon transform [4], which does not exhibit the Gibbs ringing in the same form as FT. This fact is demonstrated in Fig. 1 using a numerical simulation. To investigate the validity of this observation in-vivo, healthy canines (N=2) and healthy human volunteers (N=4; IRB approved) were imaged on a clinical 3T scanner (Siemens Verio). Two first-pass MP scans (SR-prepared FLASH) were performed at rest (>10 minutes gap) using a single-shot radial (customized) pulse sequence followed by a single-shot Cartesian (product) sequence (common parameters: FOV read = 270-300 mm; BW ≈ 800 Hz/pixel; flip angle = 12; TR = 2.4 ms; TI = 100 ms). Both scans were accelerated using rate 2 self-calibrating parallel imaging with 36-40 readouts. The customized radial sequence incorporated gradient delay correction and a 4-fold interleaving scheme combined with KWIC processing [5]. Although the readout resolution for the Cartesian scan was higher than the base resolution for the radial scheme (≈1.8 mm vs. ≈2.2 mm), the overall resolution of Cartesian (PE resolution≈2.6 mm) and radial scans were matched to within 10%.

## Results

**Figure 2:**
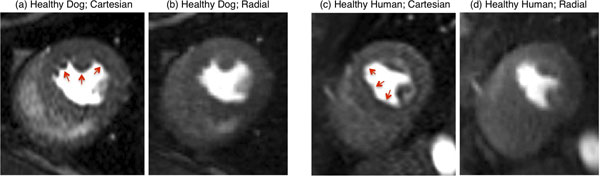
Representative in-vivo results for rest first-pass myocardial perfusion scans: (a,b) healthy canine model; (c,d) healthy human volunteer. Each pair of Cartesian/radial images corresponds to the same phase of the contrast uptake (late-LV or early-myocardial enhancement phase). The arrows in Panels (a) and (c) point to dark-rim artifacts (DRAs). However, DRAs are not seen in the projection-reconstruction (radial sampling) images in Panels (b) and (d).

Fig. 2 shows the reconstruction results for representative dog (a,b) and human (c,d) scans. Each pair of Cartesian/radial images correspond to the same phase of the contrast uptake (late-LV/early-myocardial enhancement). As is seen in the figure, Cartesian images (a,c) show DRAs (arrows pointing to hypo-intensities) in the subendocardial regions whereas the projection reconstructions (b,d) are free of dark rims (with slightly reduced CNR).

## Conclusions

Recently, a major approach for eliminating the DRAs has been to improve the spatial resolution and thereby reducing Gibbs ringing [1,6]. In this work, we demonstrated that projection imaging is inherently robust to Gibbs ringing. We conclude that an alternative strategy (besides increasing the resolution) is to employ projection imaging (radial trajectories). The presented results were limited to rest scans, although we expect the same properties to hold for stress imaging. However, to match the high resolutions achieved in advanced Cartesian schemes (e.g., [6]), rate 2 parallel imaging acceleration is not sufficient and constrained highly-accelerated reconstruction will be needed.

## Funding

Grant sponsors: American Heart Association Postdoctoral Fellowship Award 11POST7390063 (PI: B. Sharif); National Institutes of Health grants nos. NHLBI HL38698 (PI: D. Li) and NHLBI HL091989 (PI: R. Dharmakumar).
